# Perceptions of postnatal care: factors associated with primiparous mothers perceptions of postnatal communication and care

**DOI:** 10.1186/1471-2393-13-227

**Published:** 2013-12-09

**Authors:** Julie McLellan, Anita Laidlaw

**Affiliations:** 1 Medical School, University of St Andrews, Medical and Biological Sciences Building, Rm 216, St Andrews, Fife KY16 9TF, Scotland

**Keywords:** Postnatal, Communication, Satisfaction with care, Breastfeeding, Personality, Anxiety, Depression

## Abstract

**Background:**

The aim of this study was to determine whether personality and/or psychological functioning affect mothers’ perceptions of postnatal communication and their level of satisfaction with their postnatal care. Mothers’ perceptions of the communication with health professionals prenatally and during birth may be affected by their personality traits and psychological functioning and are linked to the level of satisfaction they have in their healthcare. Little is known about factors that are associated with perceptions of communication within postnatal care and the impact this may have on satisfaction with care.

**Methods:**

A cross sectional survey recruited 71 first-time mothers, who had given birth vaginally in the U.K., within the previous 12 months. The questionnaire assessed personality traits using the Big 5 Mini Marker set, levels of anxiety and depression using HADS, perceptions of communication experienced with health professionals and overall levels of satisfaction with postnatal care via the Satisfaction with Care Scale. Covariates such as demographic factors were investigated.

**Results:**

Higher ratings of communication were found to be directly correlated with higher satisfaction, as were the personality traits; agreeableness, conscientiousness and emotional stability. Depression significantly lowered participants’ ratings of communication experienced with health visitors and total satisfaction. Mothers who breastfed had significantly lower communication and satisfaction ratings. Multiple regression analysis revealed communication ratings explained 71.8% of the variance in the level of satisfaction and none of the remaining predictors significantly directly affected satisfaction ratings.

**Conclusion:**

Future research should focus on the utility of these findings for improving care for primiparous mothers.

## Background

Ensuring that new mothers are satisfied with their maternity care is vitally important as it occurs during, arguably, the biggest transition they will face in life, and can significantly impact upon their child bearing experience [1] and wellbeing of the whole family [2]. Part of maternal healthcare includes ensuring the mothers’ emotional support needs are met [2] meaning healthcare professionals are required to enquire into how mothers cope psychologically. Healthcare communication has been shown to impact on levels of patient satisfaction [3]. Within the specialty of maternity care, measures of communication, such as time spent discussing problems, have been shown to be significantly linked to satisfaction levels in prenatal care and care during labour and delivery [4], or with satisfaction with maternity care as a whole [5]. Although this finding appears to extend to the postnatal field [6], less is known, and there have been calls for more research in this area [7]. It is necessary to consider factors that may influence perceptions of communication between new mothers and healthcare professionals. Many mothers, particularly those who are having their first baby, have expectations that postnatal care will reduce their anxiety by providing them with the skills and the confidence to care for their baby [8]. However these expectations are often not met as research has shown that a sample of first-time mothers in the U.K. had a range of unmet information needs, such as knowing how frequently to breastfeed, how often to pick up their baby when crying and how often to change their nappy [9]. There are few studies which examine the interactions of personality with postnatal care satisfaction, however cancer patients with high trait anxiety and low self-esteem, were more likely than patients without these characteristics to perceive the communication they received as being insufficient and ambiguous, suggesting unmet needs [10]. Such finding, although in a different patient population, could prove to be particularly pertinent to primiparous mothers when it is considered that between 10% and 15% of postpartum women meet the criteria for postnatal depression [11]. Research has shown a link between the big five personality traits (a psychological personality inventory of openness, conscientiousness, extroversion, agreeableness and neuroticism [12]) and depression [13], with high neuroticism associated with higher levels of depression and low extraversion being associated with being less happy in life. Personality may affect communication perception through variables such as birth method. An effect of personality on preferred mode of delivery, with women with lower levels of socialisation and higher levels of monotony avoidance preferring to give birth via elective caesarean as opposed to vaginally has also been reported [14]. Therefore, evidence indicates that there could potentially be direct (via differences in perceptions of the communication occurring) or indirect (via differences to the actual communication occurring) effects of personality and psychological functioning on perceptions of communication and subsequent satisfaction with healthcare throughout pregnancy and labour. However, no previous research has examined whether such findings apply within the field of postnatal healthcare. This study therefore aims to examine the relationships between maternal personality, mental health, perceptions of the communication they receive in the postnatal period from midwives and health visitors and their satisfaction with postnatal care and to explore the relative impacts of these factors. This study attempts to answer three questions; 1) do demographic factors such as maternal age or the level of deprivation a mother lives in directly impact upon satisfaction with postnatal healthcare?, 2) does the psychological functioning and/or personality traits of those mothers directly or indirectly impact upon satisfaction with postnatal healthcare? And, 3) does the perception of the healthcare communication primiparous mothers receive during the postnatal period directly impact upon satisfaction with postnatal healthcare?

## Methods

This cross sectional survey took place between March and July 2011. First-time Mothers who had given birth vaginally in U.K. in the previous 12 months, at full term (37 weeks and over) completed the questionnaire. The questionnaire consisted of independent variables including ratings of communication (with midwives, health visitors or overall), personality traits, psychological functioning (anxiety and depression), demographic factors (such as maternal age) and additional factors (for example, time period since birth). The dependent variable was level of satisfaction with postnatal healthcare.

The postnatal period was defined as the point of discharge from hospital (or in the case of homebirths the care following the birth experience) until 12 months after birth. Participants were opportunistically recruited either online recruitment via advertisements on appropriate online forums such as ‘NETMUMS’ and ‘Mumsnet’ or via direct approach at a variety of Mother and Baby groups such as ‘Baby Sensory’ and ‘Rhyme Time’ in East and West Scotland. Mothers who were recruited online completed the questionnaire electronically whilst those recruited in person filled out a paper version. In order to determine how many participants were needed a power calculation was carried out using the statistical software “nQuery Advisor”. It was reported that for a multiple linear regression model which already includes 2 covariates with a squared multiple correlation R^2^ of 0.0500, a sample size of 69 will have 80% power to detect at α = 0.050 an increase in R^2^ 0.1500 due to including 4 additional covariates.

The big 5 personality traits were measured using the 40 item Mini Marker Set [15]. The mini-markers provide a shortened version of Goldberg’s [16] 100 item markers of the big 5 and according to Saucier [15] improve upon the specificity of Goldberg’s original inventory. Psychological functioning was measured using the 14 item Hospital Anxiety and Depression Scale (HADS) [17] which is widely applied within both research and clinical settings. It has been validated against longer established standardised questionnaires [18] such as the Beck’s Depression Inventory [19] and has been shown to be reliable within the general population [20]. Scores in each subscale (anxiety or depression) of between 8 and 10 indicate a mild case, 11–15 indicate a moderate case and 16 or above suggests a severe caseof anxiety and/or depression [21]. The Satisfaction with Care Scale was developed by Hall, Feldstein, Fretwell, Rowe & Epstein [22]. Despite being developed for use in an elderly population, it is based largely on the satisfaction with physicians conduct scale devised by DiMatteo and Hays [23]. It consists of 12 items which ask participants to rate their satisfaction with healthcare providers during the past 3 months in regard to total satisfaction, amount of contact with providers, communication behaviours of providers, humaneness of providers, technical competence of providers, and relief of worry. Due to the nature of these sub-elements, and it’s validation within an adult sample, it was used in this study [22] (Hall, Feldstein, Fretwell, Rowe & Epstein, 1990). Lubeck et al. [24] showed the overall scale to have good internal consistency with a Cronbach alpha reliability score of 0.82. In this study the Cronbach alpha was higher, at 0.935 (Additional file 1). Minor modifications were made to five Likert scale items from the widely validated General Practice Assessment Questionnaire [25] to measure participants’ perceptions of the communication they experienced with health professionals during the postnatal period. Participants completed these questions separately with regard to midwives, health visitors and ‘other’ health professionals they came into frequent contact with during the postnatal period. Participants were also asked to give an overall rating of the communication they experienced with health professionals during their postnatal care using a Visual Analogue Scale.

Some demographic questions were included such as maternal age, participants also provided postcode in order to measure deprivation in the area of residence using the unemployment rates from the Multiple Deprivation Indice [26]. Factors including the number of months since they had given birth and whether they had breastfed, and if so for how many months, were also recorded.

The data was analysed using the Predictive Analytics Software (PASW) version 18. Data was investigated for normality and descriptive statistics were conducted for all items. Results are expressed as means ± standard deviations unless otherwise stated. Statistical significance was set at P < 0.05. Means testing was used initially to determine whether demographic and additional factors and psychological functioning affected ratings of communication and satisfaction. Correlational analysis was used to assess the relationship between personality traits and ratings of communication and satisfaction, whilst hierarchical multiple linear regression analysis assessed whether communication directly or indirectly affected satisfaction with postnatal healthcare.

This study was approved by St Andrews University Medical School Ethics Committee. Informed consent was provided prior to questionnaire completion by all participants following the reading of an information sheet. The opportunity to ask questions regarding the study was also provided.

## Results

### Descriptive statistics

During the study 120 questionnaires were given out via baby groups and 40 of these were returned completed (33% response rate). In total 69 individuals accessed the survey online with 31 completing it (45% completion rate). In total 71 mothers completed the questionnaire (32 recruited online, 39 from baby groups). A summary of descriptive data for the whole sample, categorised by recruitment method, is shown in Table 1. The data was tested for normality and although some slight deviations were found these were not large enough to impact on the data, therefore the data was not transformed. There were no major differences demographically between the online sub- sample and the parent and baby group sub-sample, other than a slightly higher number of mildly depressed participant in the online sub-sample.

**Table 1:** Descriptive characteristics of the demographic and additional factors of the sample

	**Entire sample**	**Parent and baby group sub-sample**	**Online sub-sample**
	**(N = 71)**	**(N = 39)**	**(N = 32)**
**Mean age of participants (years)**	*29*	*30*	*29*
**Age range**	*Minimum age = 19*	*Minimum age = 19*	*Minimum age = 19*
	*Maximum age = 41*	*Maximum age = 41*	*Maximum age = 37*
**Unemployment rate average (%)**	*10%*	*10%*	*10%*
**Mean time period since birth (months)**	*7*	*7*	*7*
**Number of breastfeeding participants**	*55 (77%)*	*27 (69%)*	*28 (87.5%)*
**Mean breastfeeding length (months)**	*2.4*	*2.5*	*2.3*
**Number of participants with HADS depression score:**			
**Not depressed (0–7)**	*59*	*36*	*23*

### Do demographic and other descriptive factors impact upon satisfaction with postnatal healthcare?

The only demographic or additional factor which significantly impacted on either satisfaction with care or perceptions of communication was method of feeding (breastfeeding versus non-breastfeeding). Other factors, such as time since giving birth, had no statistically significant association with satisfaction with care or perceptions of communication.

**Figure 1:**
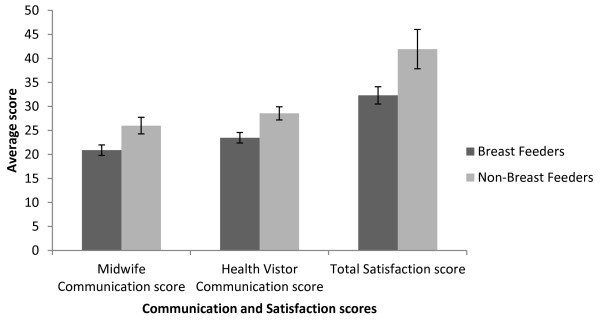
**Mean total satisfaction with postnatal care and communication ratings for breastfeeding mothers and those who were not breastfeeding.** Error bars represent standard errors.

Independent-samples t-tests revealed that compared to breastfeeding mothers (n = 55), non-breastfeeding mothers (n = 16) had higher mean satisfaction with care scores (breastfeeding mothers M = 32.31, SD = 13.37, non-breastfeeding mothers M = 41.94, SD = 16.33); t (69) = -2.410, p = .019, (two-tailed). There was a significant difference in perceptions of midwife communication between mothers who had breastfed (M = 20.89, tailed) with similar results observed for health visitors communication (mothers who breastfed M = 23.47, SD = 8.10, non-breastfeeding mothers (M = 28.56, SD = 5.48); t (69) = - 2.357, p = < .021, (two-tailed), see Figure 1. There was no impact of breastfeeding on overall communication ratings.

### Does the psychological functioning and/or personality traits of mothers’ impact upon satisfaction with postnatal healthcare?

**Figure 2:**
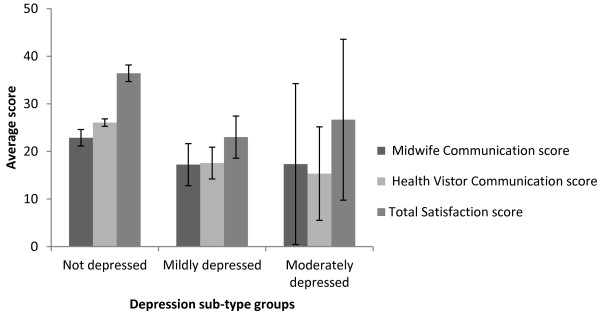
**Mean total satisfaction with postnatal care and communication ratings grouped by depression sub-types.** Error bars represent standard errors.

Participants were divided into 3 groups based on their HADS depression score and a one- tailed, one-way between groups analysis of variance (ANOVA) revealed there was a statistically significant difference in communication perception at the p = .001 level between the three depression sub-groups: F (2, 4.40) = 8.3, p = .001 between groups. Post-hoc comparisons using the Gabriel test indicated that the mean health visitor communication score for the non-depressed participants (M = 26.17, SD = 5.99, n = 59) was significantly higher than either mildly depressed (M = 17.55, SD = 10.01, n = 9) or moderately depressed participants (M = 15.33, SD = 17.04, n = 3), see Figure 2. This effect was not observed for perceptions of communication with midwives or on overall communication ratings. Using the same method of analysis, a statistically significant difference at the p = .021 level in satisfaction with healthcare scores for the three depression sub-groups was noted: F (2, 67) = 4.2, p = .05. Post-hoc comparisons using the Gabriel test indicated that only significant difference was between the mean satisfaction score for the non-depressed participants (M = 36.63, SD = 13.52) and the mildly depressed participants (M = 23.00, SD = 13.29), see Figure 2. Similar analyses for the anxiety subscale of HADS resulted in no significant differences being observed.

SD = 8.12) and those who had not breastfed (M = 26, SD = 6.93); t (69) = -2.283, p = .026, (two- Pearson correlational analysis showed that conscientiousness and emotional stability had a medium association with overall communication rating (p = .005 and p = .005 respectively) whilst agreeableness had a significant but low association (p = .01) [27] see Table 2. Conscientiousness and emotional stability also had a consistent association (p = .005 and p = .002 respectively) with midwives’ communication ratings, whilst agreeableness again had a low association (p = .039). Agreeableness and emotional stability had low associations (p = .015 and p = .029 respectively) and conscientiousness medium association (p = .002) with health visitor communication ratings, Table 2. Further, agreeableness (p = .004), conscientiousness (p = .01) and emotional stability (p = .001) all had a medium association with satisfaction score, see Table 2. Extraversion and openness were not found to be correlated with any of the measures of communication or satisfaction.

**Table 2:** Correlations between personality traits and communication and satisfaction ratings and correlations between communication measures and total satisfaction

		**Overall communication**	**Heath visitor communication**	**Midwives communication**	**Total satisfaction**
**Agreeableness**	**Pearson correlation**	.277^**^	.259^*^	.245^*^	.339^**^
	**Sig. (1-tailed)**	.010	.015	.039	.004
	**N**	71	71	71	71
**Conscientiousness**	**Pearson correlation**	.303^**^	.331^**^	.328^**^	.305^**^
	**Sig. (1-tailed)**	.005	.002	.005	.010
	**N**	71	71	71	71
**Emotional stability**	**Pearson correlation**	.306^**^	.226^*^	.363^**^	.386^**^
	**Sig. (1-tailed)**	.005	.029	.002	.001
**Satisfaction**	**N**	71	71	71	71
	**Pearson correlation**	.716**	.672**	.761**	
	**Sig. (1-tailed)**	.000	.000	.000	
	**N**	71	71	71	

*Sig at P = 0.05 level, **Sig at P = 0.01 level.

### Do the perceptions of the healthcare communication primiparous mothers receive during the postnatal period directly impact upon satisfaction with postnatal healthcare?

Relationships between mothers’ perceptions of communication experienced and total satisfaction were investigated using Pearson correlations (displayed in Table 2). There was a strong, positive correlation between midwife, health visitor and overall communication and total satisfaction, (midwife: r = .761, n = 71, p < .0005, health visitor: r = .672, n = 71, p < .0005, overall: r = .716, n = 71, p < .0005) with high levels of perceived communication associated with high levels of total satisfaction.

In order to investigate whether satisfaction with postnatal care was directly or indirectly affected by variables, a four-step hierarchical multiple linear regression (Table 3) was carried out using variables that were previously shown to significantly impact upon communication and satisfaction. Communication ratings were entered at step 1, explaining 71.8% of the variance in total satisfaction. Entry of HADS depression sub-type at step 2 did not change the total variance explained by the model as it remained at 71.% as a whole, F (4, 66) = 41.943, p < .001 with R squared change = .000, F change (1, 66) = .032, p > .05. In the third model feeding method was added and the variance explained was 72.5%, F (5, 65) = 34.345, p < .001, accounting for an additional 0.8% of the variance in total satisfaction, after controlling for communication ratings and HADS depression sub-type, R squared change = .008, F change (1, 65) =1.833, p > .05. Agreeableness, emotional stability and conscientiousness were added at the final model and the overall variance explained was 74%, F (8, 62) =22.048, p < .001, accounting for an additional 1.4% of the variance in total satisfaction, after controlling for communication ratings, HADS depression sub-type and feeding method, R squared change = .014, F change (3, 62) =1.152, p > .05. Therefore in the final model, only the communication ratings controlled for in the first model were statistically significant, with the overall communication ratings having recorded a higher beta value (beta = .365) than the midwife (beta = .294) and health visitor (beta = .259) communication ratings.

**Table 3:** 4-step hierarchical multiple linear regression

**Predictors**	**Step 1**			**Step 2**			**Step 3**			**Step 4**		
	**B**	**SE B**	**β**	**B**	**SE B**	**β**	**B**	**SE B**	**β**	**B**	**SE B**	**β**
Constant	-7.375	3.459		-7.729	4.009		-10.202	4.383		-20.953	8.664	
Overall communication	.232	.053	.369***	.233	.054	.370***	.242	.054	.385***	.230	.054	.365***
Midwife communication	.621	.176	.346***	.618	.178	.345***	.578	.179	.322**	.527	.184	.294***
Health visitor communication	.524	.157	.283***	.535	.170	.289**	.488	.172	.263*	.480	.176	.259*
HADS depression sub-group				.376	2.102	.013	-.103	2.119	-.004	.242	2.144	.008
Feeding method							3.264	2.411	.094	3.636	2.495	.105
Agreeableness										1.109	1.004	.080
Emotional stability										-.195	.848	-.017
Conscientiousness										1.029	.944	.083

Note: R² = .72 for Step 1, adjusted R²= 0 for Step 2 (*p > .05),* adjusted R²= .008 for Step 3 ((*p > .05),* adjusted R²= .014 for Step 3 (*p* > .05) *p < .001, **p < .005, ***p < .01.

## Discussion

### Representativeness of the sample

The average age a woman in the U.K. has her first child is 29 [28], the same as found in this study. The deprivation level of participants, assessed by average unemployment rate in this study was also similar to the U.K. average (survey participants = 10%, U.K. average = 8. 4% [29]. The high percentage of breastfeeding mothers (73%) is not surprising given the high level who attempt breastfeeding. In this study however, breastfeeding on average it lasted for approximately 2 and a half months in this study, longer than the previously recorded six- weeks [2]. The levels of mild / moderate depression within the study sample (17%) are, reflective of the landmark meta-analysis carried out by O’Hara & Swain [11] which revealed 10- 15% of postpartum women meet the criteria for postnatal depression. Therefore, overall it would appear that the sample of first time mothers who completed this survey were comparable, but in no way a complete representation of, the general population in the U.K.

### Impact of breastfeeding

Mothers who breast fed their baby were less satisfied with the care they received and rated the communication they experienced with both midwives and health visitors lower than those who did not breastfeed. When feeding method was added into the regression model the effect of health visitor and midwife communication on satisfaction with healthcare was reduced, showing an indirect effect of feeding choice on total satisfaction. These findings highlight the importance of communication concerning infant feeding. An NCT postnatal care report [2] reported that 8 out of 10 first-time mothers intend to breastfeed, whilst other work has shown that 4 in 10 of mothers stop breastfeeding within 6 weeks of giving birth owing to a lack of relevant information and support [30]. As breastfeeding has substantial benefits for both mother and infant [31], these results are concerning.

The National Institute for Health and Clinical Excellence guidelines suggest mothers should be asked about their breastfeeding experiences at every contact with health professionals [32] so health professionals are aware of the need for communication in relation to breastfeeding. Clearly however, continual communication does not necessarily result in effective communication [2]. The present study highlights the impact that discontent regarding breastfeeding communication has on satisfaction with postnatal care. The views of these mothers is particularly concerning given that mothers who are unsuccessful in breastfeeding are at risk of developing feelings of failure, which may ultimately lead to postnatal depression [33].

### Impact of depression

Depression was shown to have a direct effect on health visitor communication ratings and total satisfaction. This confirms work that illustrated women suffering with postnatal depression were significantly less satisfied with their postnatal care compared to those who were not depressed [34]. There are several putative reasons for this finding; such as more frequent use of services and therefore greater opportunity to evaluate care [34]. Alternatively, depression has a greater influence on quality of life than anxiety [35] and therefore may result in reduced satisfaction with care due to reduced overall satisfaction. Finally, research has also shown that people who are depressed are more likely than non-depressed individuals to perceive neutral communication as being negative [36]. Care must be taken when extrapolating these results due to the slightly higher numbers of mildly depressed within the online sub-sample compared to the baby group sub-sample and further work is required to determine whether any of these postulated theories correctly describes the route of impact of depression on communication ratings and satisfaction with postnatal care.

### Impact of personality

Given the impact that depression has on communication and satisfaction, it is not surprising that there was a positive correlation with emotional stability, supporting previous findings [13]. Given the limited previous literature of any association between extraversion and patient perceptions of health care communication [37], the lack of any significant relationship in this study was unremarkable. Further, due to the work carried out by other researchers [38,39] it is also not surprising that individuals with higher levels of agreeableness have higher levels of satisfaction and perceive the communication they receive as being better. However the current study extends these findings within the postnatal field and suggests a need for health professionals to utilise information regarding personality to tailor communication and healthcare. This issue could be examined further by a more detailed analysis of communication utterances between health professionals and first time mothers to establish the importance of any link between personality and unmet needs, particularly emotional needs, for example using the Verona cues and concerns coding system [40].

### Impact of communication on satisfaction

The results of this investigation confirm that the effect of poor communication on satisfaction with care noted in doctor – patient relationships [41] extends into the field of postnatal care, as suggested by previous researchers who have reported that various aspects of interactions with health care providers, such as concerns being taken seriously or health care providers appearing rushed, were predictive of satisfaction with early postnatal care [4-6]. Since communication is regarded as a key component of a midwife’s role by the Nursing and Midwifery Council [42] the importance of it is obviously well acknowledged by suchhealthcare professionals, however, a substantial proportion of women are currently not happy with the communication they receive [2]. This is particularly concerning given that our analysis revealed that the majority of the variability (71.8%) in satisfaction ratings could be explained by communication perceptions. It is clear from the current study that, although personality traits, breastfeeding and depression are significant factors, collectively they only represent approximately 2% of the variance in satisfaction scores highlighting the vital importance of communication in satisfaction with postnatal care.

### Limitations

Care must be taken when considering these findings. Although the data suggests the current sample were fairly representative of the population of primiparous mothers in the U.K, the sample is relatively small (although statistical power was achieved) and some demographic information which may be relevant, such as level of education, was not collected. The sample is also limited by recruitment methods, via postnatal support groups or online social media forums, with the majority of participants being located in central Scotland. Whilst several attempts were made to reach as wide a variety of mothers from different socio-economic backgrounds, a significant proportion of the data gathered came from mothers attending privately run classes for their baby, therefore the results should be viewed with this in mind. For example, the above average length of breastfeeding is indicative of sampling bias [43].

Participants also received postnatal care from different health boards and therefore despite the use of a Maternity Pathways for Care system, there may be variations in the care received [2]. This ad-hoc approach highlights the additional differences women can experience in their care. It is also important to recognise that the term “postnatal” is a general term which can refer to a variety of encounters which can influence a mother’s perceptions of her healthcare experiences following the birth of her baby. Therefore it is necessary to highlight that this paper merely identified potential generalities which require further work to identify the specific mechanisms by which they operate. This is particularly pertinent when considering the issue of the interrelationship between the midwife and health visitor communication ratings as no details such as the number and nature of incidences of contact were studied. The findings relating to personality need to be interpreted with the same caution, and more research is required to understand the basis of the associations highlighted in this study between personality, healthcare and communication. It may have been useful to have recorded participants’ ethnicity as previous research has shown that those from ethnic minorities have lower levels of healthcare satisfaction [44], however this data was not recorded in the present study. Although the satisfaction questionnaire used was validated with an adult sample it was predominantly used as it fitted the specification of the study’s outline. The fact it had not been validated within the current population is however, acknowledged as a limitation of this research.

## Conclusions

**Figure 3:**
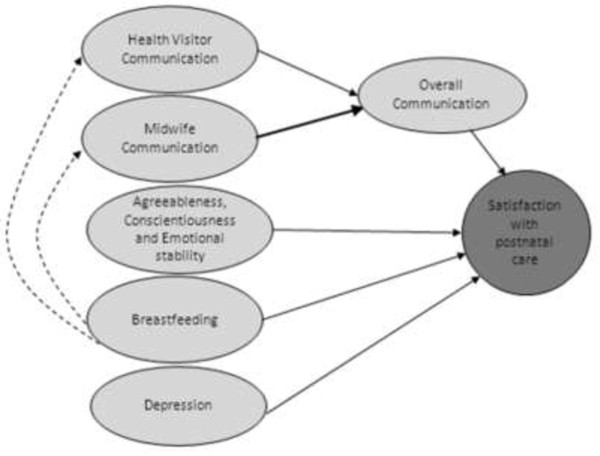
Diagram illustrating the key relationships from the study.

Overall, this novel study highlights general issues which should be subjected to more detailed research with the aim of elucidating methods of action to reduce dissatisfaction in postnatal care. This study provides a small but significant insight into factors that are associated with satisfaction with postnatal care for primiparous mothers, and the relative magnitude of any potential influence. It did not set out to investigate specific factors relating to particular aspects of postnatal care; however it highlights general areas relating to communication, feeding, mental wellbeing, and personality which potentially affect mothers’ satisfaction levels with postnatal care and the potential routes of impact, see Figure 3. Future research should focus on how this information can be further investigated to improve the provision of effective care for primiparous mothers.

## Competing interests

The authors declare that they have no competing interests.

## Authors’ contributions

JM contributed substantially to the design of the study, carried out data collection and analysis and was primarily responsible for drafting the manuscript. AL was involved in designing the study and commented on drafts of the manuscript. Both authors read and approved the final manuscript.

## Authors’ information

Julie McLellan is a St Andrews University MSc Health Psychology graduate and is currently a Trainee Clinical Associate in Applied Psychology. Anita Laidlaw is currently a Senior Teaching Fellow and Convenor of communication skills at the Medical School, University of St Andrews, UK. Her current research interests are psychological and cognitive factors affecting communication and pedagogy.

## Pre-publication history

The pre-publication history for this paper can be accessed here:

http://www.biomedcentral.com/1471-2393/13/227/prepub

## Supplementary Material

Additional file 1 Cronbach Alpha (raw data) for Patient Satisfaction Questionnaire. Click here for file
