# Supplementary material for: Perceptions of postnatal care: factors associated with primiparous mothers perceptions of postnatal communication and care
Source: BMC Pregnancy Childbirth. 2013 Dec 9;13:227. doi: 10.1186/1471-2393-13-227 (PMC4029156; doi:10.1186/1471-2393-13-227)
Supplement: Additional file 1 — Cronbach Alpha (raw data) for Patient Satisfaction Questionnaire. [file 1471-2393-13-227-S1.pdf]

```
GET
  FILE='C:\Users\ahl1\Documents\Teaching\HP Masters\projects\2010-2011\Juli
e McLellan 2010-2011\data and analysis\DATA SHEET 19th july final copy.sav'
.
DATASET NAME DataSet1 WINDOW=FRONT.
RELIABILITY
  /VARIABLES=q0045_0001 q0045_0002 q0045_0003 q0045_0004 q0045_0005 q0045_0
006 q0045_0007 q0045_0008 q0045_0009 q0045_0010 q0045_0011 q0045_0012
  /SCALE('ALL VARIABLES') ALL
  /MODEL=ALPHA.
```

Reliability

```
[DataSet1] C:\Users\ahl1\Documents\Teaching\HP Masters\projects\2010-2011\J
ulie McLellan 2010-2011\data and analysis\DATA SHEET 19th july final copy.s
av
```

Scale: ALL VARIABLES

Case Processing Summary

		N	%
Cases	Valid	71	40.3
	Excluded[a]	105	59.7
	Total	176	100.0

a. Listwise deletion based on all variables in the procedure.

Reliability Statistics

Cronbach's Alpha	N of Items
.935	12
